# An ultrasensitive electrogenerated chemiluminescence-based immunoassay for specific detection of Zika virus

**DOI:** 10.1038/srep32227

**Published:** 2016-08-24

**Authors:** Dhiraj Acharya, Pradip Bastola, Linda Le, Amber M. Paul, Estefania Fernandez, Michael S. Diamond, Wujian Miao, Fengwei Bai

**Affiliations:** 1 Department of Biological Sciences, University of Southern Mississippi, Hattiesburg, MS 39406, USA; 2 Department of Chemistry and Biochemistry, University of Southern Mississippi, Hattiesburg, MS 39406, USA; 3 Departments of Medicine, Molecular Microbiology, Pathology and Immunology, Washington University School of Medicine, St. Louis, MO 63110, USA.

## Abstract

Zika virus (ZIKV) is a globally emerging mosquito-transmitted flavivirus that can cause severe fetal abnormalities, including microcephaly. As such, highly sensitive, specific, and cost-effective diagnostic methods are urgently needed. Here, we report a novel electrogenerated chemiluminescence (ECL)-based immunoassay for ultrasensitive and specific detection of ZIKV in human biological fluids. We loaded polystyrene beads (PSB) with a large number of ECL labels and conjugated them with anti-ZIKV monoclonal antibodies to generate anti-ZIKV-PSBs. These anti-ZIKV-PSBs efficiently captured ZIKV in solution forming ZIKV-anti-ZIKV-PSB complexes, which were subjected to measurement of ECL intensity after further magnetic beads separation. Our results show that the anti-ZIKV-PSBs can capture as little as 1 PFU of ZIKV in 100 μl of saline, human plasma, or human urine. This platform has the potential for development as a cost-effective, rapid and ultrasensitive assay for the detection of ZIKV and possibly other viruses in clinical diagnosis, epidemiologic and vector surveillance, and laboratory research.

Zika virus (ZIKV) is a member of the *Flavivirus* genus of the *Flaviviridae* family, which includes other globally important pathogens, such as West Nile (WNV), dengue (DENV), and yellow fever viruses^1^,^2^. Recent outbreaks of ZIKV have linked this previously neglected virus to the development of severe fetal abnormalities including spontaneous abortion, stillbirth, microcephaly, and Guillain-Barré syndrome (GBS), a neurological disorder^3^. Autochthonous transmission of ZIKV infection has been reported in Asia, Africa, Micronesia, and Latin America with increasing descriptions of travel related cases worldwide^4^,^5^,^6^,^7^,^8^. Since the *Aedes* species of mosquitoes that transmit ZIKV circulate globally, there is a significant risk of ZIKV spread worldwide^9^,^10^. Thus, ZIKV is considered as a priority pathogen with a great public health threat^11^,^12^. The World Health Organization has estimated that as many as four million people could be infected in American countries^13^. Currently, there is no approved therapy or vaccine against ZIKV.

Similar to DENV, WNV and chikungunya virus (CHIKV), ZIKV infection in humans can result in a range of clinical signs and symptoms including fever, rash, joint pain, and conjunctivitis, which poses a challenge for differential diagnosis of these viral infections as they often are co-transmitted in the same areas^14^,^15^. In addition, ZIKV has been detected in semen, urine, and saliva^16^,^17^,^18^, suggesting a potential for transmission through routes other than mosquito bite, such as sexual contact^19^,^20^,^21^. Therefore, a highly sensitive and specific method for detection of ZIKV is urgently needed to facilitate efficient case management, surveillance, and implementation of control programs. Current methods for detection and diagnosis of ZIKV infections include nuclear acid-based assays, such as quantitative polymerase chain reaction (QPCR), and antibody-based assays, such as enzyme-linked immunosorbent assay (ELISA) or neutralization tests^22^,^23^,^24^. Since the conventional nuclear acid-based assays require expensive reagents and instruments, and ELISAs are time consuming and have specificity concerns^25^,^26^, a rapid and simple-to-use methodology with high sensitivity, reliability, and cost effectiveness is needed^27^,^28^.

Electrogenerated chemiluminescence (ECL) is a phenomenon of light emission by an excited state of species generated at electrodes that undergo high-energetic electron-transfer reactions^29^. The emitted light can be measured easily by simple instrumentation^29^,^30^, thus providing an efficient sensor for rapid and simple-to-use devices for separation, detection, and quantification of a diverse range of analytes in a small quantity (up to femtomolar scale) of starting sample. Applications of ECL-based detection have been demonstrated successfully in a wide range of immunological and molecular assay systems including clinical diagnosis^30^,^31^,^32^, biosensing^33^,^34^,^35^, drug assays^36^, food quality analysis^37^,^38^, environmental monitoring^39^,^40^, and forensic chemistry^41^,^42^,^43^. Several strategies have been developed to optimize and amplify ECL detection signals. For example, we previously reported that the polystyrene beads (PSBs) could be loaded with several billions of water-insoluble homemade Ru(bpy)_3_[B(C_6_F_5_)_4_]_2_ (bpy = 2,2′-bipyridine) ECL labels. This step increased the ratio of ECL label to bioanalyte and amplified ECL signals by several order of magnitudes^30^,^44^. By conjugating ECL label-loaded PSBs with specific antibody or antigen, a highly sensitive detection platform of diverse repertoire of analytes can be achieved.

In this paper, we report a highly sensitive and specific ECL-based immunoassay for detection and quantification of ZIKV in biological fluids with a limit of detection of 1 plaque-forming unit (PFU) in 100 μl of samples. This study provides an essential proof-of-concept that ECL-based immunoassays can be developed for the detection and quantification of viruses.

## Results

### Optimization of rubrene-based ECL-detection assay

**Figure 1:**
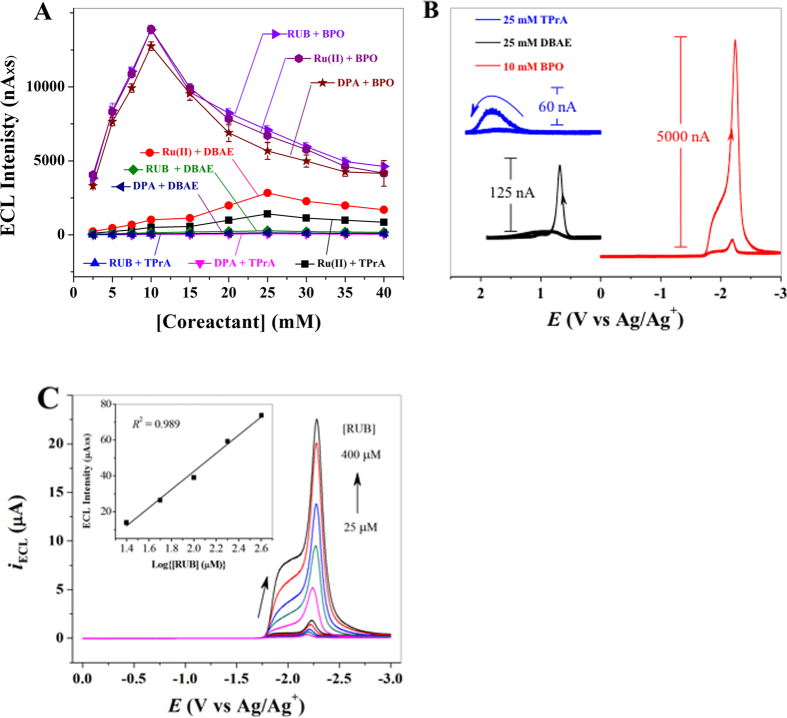
Optimization of ECL-detection conditions. Optimal ECL-detection conditions were determined by using nine different ECL-emitters/coreactant combinations. (**A**) Integrated ECL-intensity of constant concentration (25 μM) of three different ECL-emitters (RUB, Ru(bpy)_3_^2+^, and DPA) with various concentrations of three different co-reactants (BPO, DBAE, and TPrA). (**B**) ECL-intensity of 25 μM RUB as a function of potential for different co-reactants (concentrations indicated in figure). (**C**) ECL-response profiles of different concentrations of RUB with 10 mM BPO as co-reactant. A linear relationship between the integrated ECL intensity and the logarithm of RUB concentration is shown in inset. All ECL-intensity measurements were carried out in MeCN solution containing 0.10 M TBAP supporting electrolyte after degassed with N_2_ for 7 min at a 2-mm diameter Pt electrode with a scan rate of 100 mV/s. ECL-intensity of each sample was measured for three times. All experiments were performed in duplicates and repeated at least one time.

Although a variety of ECL emitters are available, only those with extremely low water solubility and high ECL efficiencies are candidates when PSBs are used as carriers of ECL emitters. This is because leached ECL emitters from PSBs during a series of chemical and biochemical interactions or conjugations as well as washing processes could contaminate the reaction system, resulting in an undesirable background. ECL emitters with a constant and maximum loading capacity in PSBs will ensure that, in subsequent processes, strong ECL signals are produced reproducibly. On the basis of the above criteria, Ru(bpy)_3_[B(C_6_F_5_)_4_]_2_, 9,10-diphenyl anthracene (DPA), and 5,6,11,12 tetraphenyl trtracene (rubrene, RUB) were selected for initial testing. Because experimental conditions for ion annihilation ECL generation are strict (e.g., wide potential window, dry electrolyte solution, and no oxygen), and detection of viruses generally involve aqueous solutions, coreactant ECL systems^45^ are an optimal choice. Three most commonly used ECL coreactants, namely 2-(dibutylamino) ethanol (DBAE) and tri-*n*-propylamine (TPrA) for anodic ECL, and benzyl peroxide (BPO) for cathodic ECL, were coupled with the three ECL emitters to investigate their ECL responses. As shown in Fig. 1A, among the three tested coreactants, BPO yielded the most efficient ECL intensities for all three ECL emitters with nearly the same ECL intensity vs. [coreactant] profiles. Maximum ECL intensities appeared at 10 mM BPO, which likely is explained by the two opposing roles of BPO has during the ECL generation. While BPO is needed for the production of BPO^−•^, ^3^R* (R = Ru(bpy)_3_^2+^, DPA, or RUB), and R^+•^, which is favorable to ECL generation, BPO can react with the electrogenerated R^−•^, which reduces the chance of the ion annihilation reaction of R^+•^ + R^−•^ → R*, and diminishes ECL generation. Smaller ECL intensities were observed across the entire concentration range of BPO when DBAE and TPrA were used as coreactants. This could be ascribed to the relative instability of R^+•^ cation radicals in the reaction media as well as the insufficient reducing capacity of TPrA^•^ and DBAE^•^ free radicals. Although the RUB/BPO and the Ru(bpy)_3_^2+^/BPO systems show similar ECL intensities, we chose the RUB/BPO system for virus detection, because RUB is insoluble in water and commercially available, whereas the synthesis of Ru(bpy)_3_[B(C_6_F_5_)_4_]_2_ was nontrivial and expensive^44^. Figure 1B shows the ECL intensity of 25 μM RUB as a function of potential at the optimal concentrations of coreactants TPrA, DBAE, and BPO, where the RUB/BPO system displays an ECL intensity that is approximately 50 and 105 times of that of the RUB/DBAE and RUB/TPrA systems, respectively. An excellent linear relationship between the ECL intensity and the logarithm of RUB concentration in the presence of 10 mM BPO is illustrated in Fig. 1C, which provides a basis for ECL based quantitative analysis.

### Assay design, ECL-label loading and antibody conjugation of polystyrene and magnetic beads

**Figure 2:**
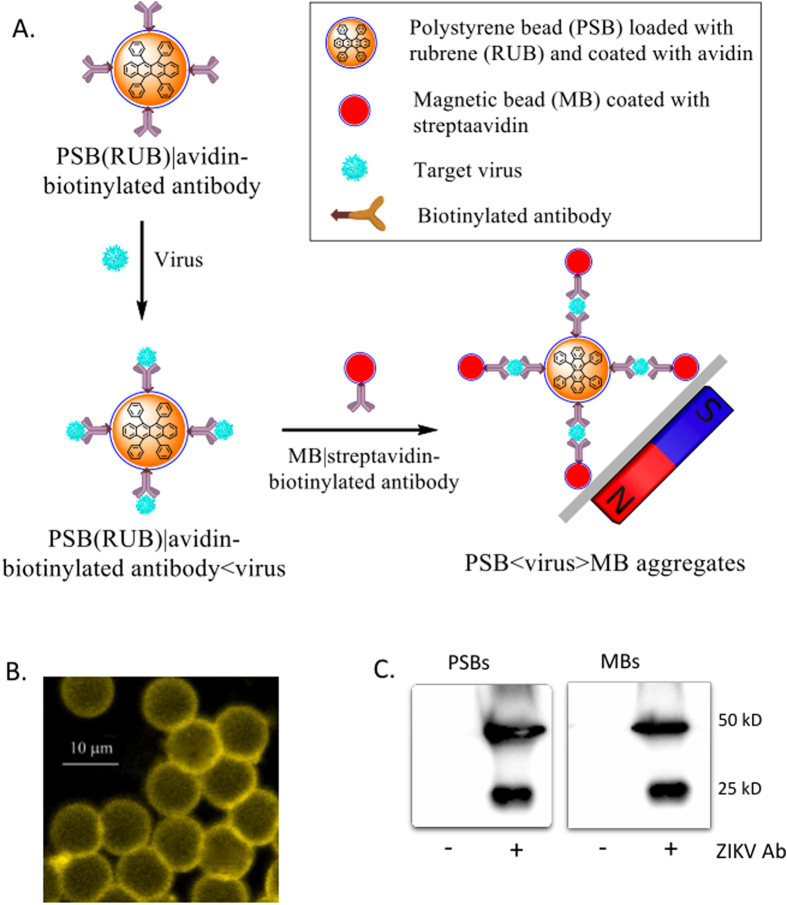
Assay design and preparation of immuno-conjugated and ECL-loaded beads. (**A**) Diagram of ECL-based immunoassay for detection of viruses. (**B**) Image of rubrene loaded PSBs (golden yellow, right) taken under UV-light (385 nm excitation) in a confocal microscope. (**C**) Antibody conjugation to PSBs and MBs was analyzed by immunoblotting assay. All experiments were performed in duplicates and repeated at least one time.

A scheme of the ECL-based strategy for detection of viruses is shown in Fig. 2A, which is developed based on previous studies^30^,^39^,^44^ with a more sensitive RUB/BPO sensing system. Briefly, the loading of ECL-labels (e.g., RUB) to PSBs amplifies the signal intensity several orders of magnitudes, generating a highly sensitive detection system. Subsequent conjugation of ECL label loaded PSBs with virus-specific antibody allow specific binding of virus particles to the PSBs surface. By using magnetic beads (MBs) conjugated with virus-specific antibody as a capture element, virus bound to ECL-label loaded PSBs can be separated magnetically and subjected to measurement of ECL-intensity after the dissolution of the PSB < virus > MB aggregates in MeCN, the latter signal is proportional to the amount of virus present in the samples. To develop a specific and highly sensitive ECL-based immunoassay for detection of ZIKV, we loaded PSBs with RUB by immersing the beads into a saturated solution of RUB. These PSBs, designated as PSB(RUB), had a typical loading capacity of ∼3.3 × 10^9^ RUB molecules per bead, which was estimated on the basis of the ECL data obtained from the PSB(RUB) dissolved in MeCN and a set of standard RUB solutions using BPO as a coreactant. ECL label loading of PSBs with RUB was confirmed with a fluorescence microscope, which shows that PSB(RUB) beads glow as intense yellow color when exposed to UV light (Fig. 2B). No such signal was detected from unloaded (bare) PSBs (data not shown). Subsequently, we covalently attached avidin to the surface of the PSB(RUB) beads via amide bonding. Avidin conjugated PSB(RUB) beads then were mixed with a biotinylated monoclonal antibody (mAb ZV-2) that is specific to ZIKV and does not recognized closely related flaviviruses, including DENV (data not shown). This step generated an antibody-conjugated PSB(RUB) (abbreviated as anti-ZIKV-PSB in following text) for specific detection of ZIKV. In addition, we conjugated magnetic beads (MBs) with ZV-2 mAb through biotin-streptavidin conjugation (anti-ZIKV-MB), which can serve as a capture element for magnetically separating ZIKV bound to PSB(RUB) in subsequent ECL-intensity measurements. Conjugation of ZV-2 to PSB (Fig. 2C, left) and MB (Fig. 2C, right) was confirmed by Western blotting. We next studied the ECL behavior of anti-ZIKV-PSB complexes. These complexes exhibited a stable and consistent ECL response similar to those shown in Fig. 1C for RUB/BPO solutions, suggesting no alteration in ECL behavior of RUB after loading to PSB and antibody conjugation. Taken together, these data suggest that the anti-ZIKV-PSB complexes serve as both target binding reagent and ECL signal generator, and are suitable to capture ZIKV from solution.

### Anti-ZIKV-PSB capture of ZIKV

**Figure 3:**
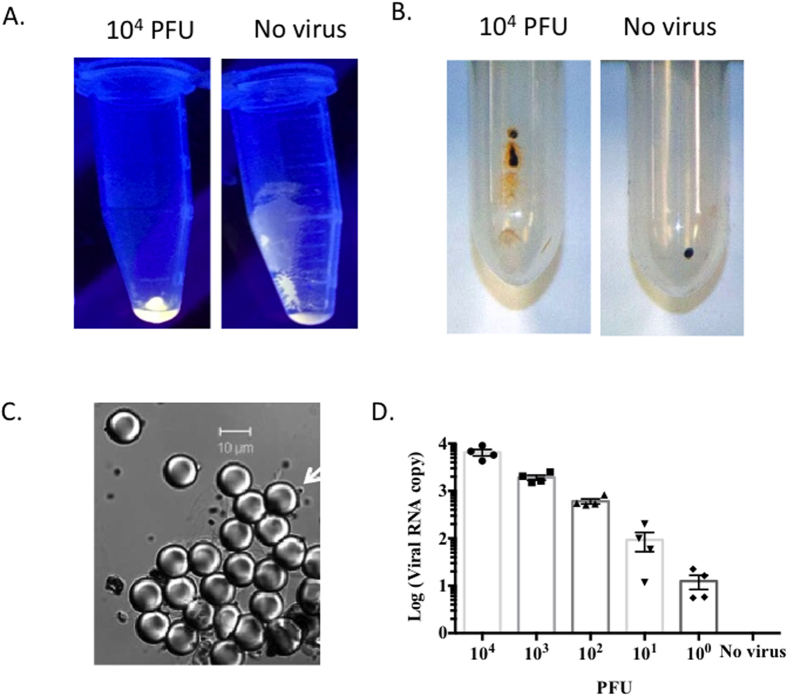
Immuno-conjugated and ECL label loaded polystyrene beads capture ZIKV. (**A**) Photograph of a microfuge tubes after addition of ZIKV containing samples to ZV2-PSB(RUB), showing the aggregation of PSBs at the bottom of tube in the presence of ZIKV. (**B**) Photograph of a microfuge tubes after magnetic separation showing the free MBs (right), and binding of PSBs to MBs (left) in the presence of ZIKV. (**C**) A phase-contrast image of PSB < ZIKV > MB aggregates showing the binding of anti-ZV2-MB (1 μm diameter, arrow) to the surface of anti-ZV2-PSB (10 μm diameter) in the presence of ZIKV. (**D**) PSB < ZIKV > MB aggregates that were obtained from samples containing different PFUs of ZIKV were analyzed by RT-qPCR assay to quantify copy number of ZIKV envelope gene. All experiments were performed in duplicates and repeated at least one time.

To test whether anti-ZIKV-PSB can capture ZIKV, 10^4^ PFU of ZIKV (strain PRVABC59) in 0.1 M PBS (pH 7.4, 100 μl) containing 2% BSA were incubated with anti-ZIKV-PSB (40 μl) for 45 min in a Dynal mixer to form ZIKV-anti-ZIKV-PSB complexes. These complexes were washed twice (8,000 g, 3 min) with PBS, re-suspended in 100 μl PBS containing 2% BSA, then reacted with 10 μl of capture element (anti-ZIKV-MBs) for 45 min in a Dynal mixer form PSB < ZIKV > MB aggregates, which were separated by using Dynal magnetic separator. After centrifugation, some of the PSBs in the control samples (without virus) attach on the wall of the microfuge tube. In comparison, after addition of viruses followed by centrifugation, the virus-PSB complexes settled down at the bottom of microfuge tube (Fig. 3A), indicating that the ZIKV causes aggregation of anti-ZIKV-PSBs. In addition, after magnetic separation, the beads that bound the PSBs can be observed in the wall of tube (Fig. 3B), suggesting the formation of PSB < ZIKV > MB aggregates in the presence of ZIKV. As shown in a micrograph (Fig. 3C), anti-ZIKV-PSB complexes undergo aggregation in the presence of ZIKV and anti-ZIKV-MBs. To detect and quantify the ZIKV bound to PSB < ZIKV > MB aggregates, we used different amounts of ZIKV (0 to 10^4^ PFU) to form aggregates, which were separated magnetically and subjected to extraction and RT-qPCR analysis of ZIKV RNA. The PCR results confirmed that ZIKV bound to PSB < ZIKV > MB aggregates validating the efficiency of ECL-based assay to capture ZIKV (Fig. 3D). Collectively, these results suggest that conjugation to ECL label loaded PSBs does not alter the specificity of anti-ZIKV antibody, suggesting that this system can be used to selectively capture the target ZIKV particles.

### Anti-ZIKV-PSB specifically detects ZIKV in a highly sensitive manner

**Figure 4:**
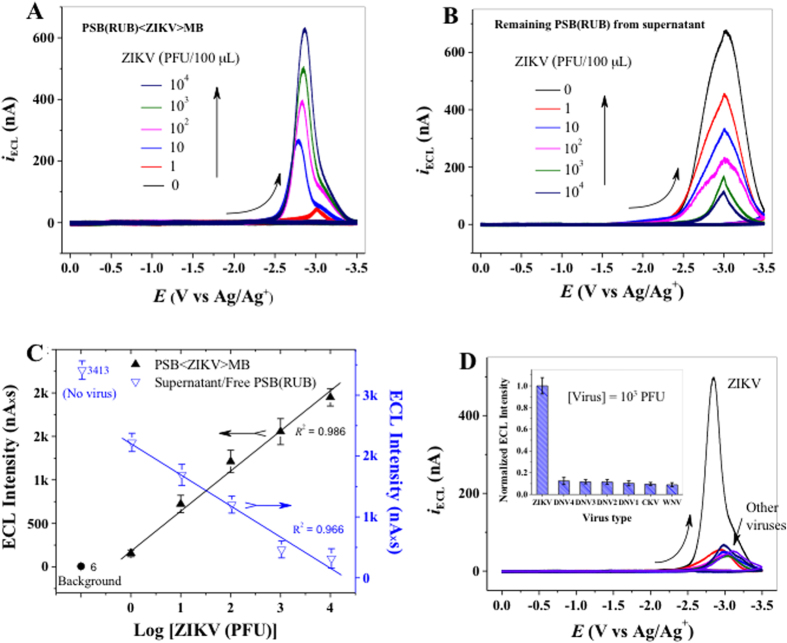
Anti-ZIKV-PSB specifically detects ZIKV in a highly sensitive manner. Samples containing different amounts of ZIKV (0–10^4^ PFU) were prepared in PBS containing 2% BSA and allowed to form PSB < ZIKV > MB complexes by reacting with ZV2-PSB(RUB) and ZV2-MB. PSB < ZIKV > MB complexes were separated magnetically and subjected to ECL-intensity measurement. (**A**) ECL-response curves of PSB < ZIKV > MB aggregates obtained from samples containing different PFUs of ZIKV in PBS. (**B**) ECL-response of free (unliganded) PSB(RUB) that remained in solution after magnetic separation. (**C**) A calibration curve of mean ECL intensity of magnetically separated PSB < ZIKV > MB aggregates (upward line, black) and free PSB(RUB) (downward line, blue) that remained in solution after magnetic separation. (**D**) ECL response of PSB < ZIKV > MB aggregates obtained from samples containing related (DENV and WNV) or unrelated (CHIKV) viruses (10^3^ PFU) prepared in PBS containing 2% BSA. Sample containing no virus was used as a control to determine background signal (~0, see Fig. 4C). All experiments were performed in duplicates and repeated at least one time.

To detect ZIKV, anti-ZIKV-PSBs were mixed with different amounts of ZIKV (0 to 10^4^ PFU) diluted in PBS with 2% BSA. The resulting virus-PSB conjugates were washed to remove unbound viruses and re-suspended. Virus-PSB conjugates then were allowed to react with the capture element (i.e., anti-ZIKV-MB) to form sandwich-type PSB < ZIKV > MB aggregates, which were magnetically separated from the reaction medium. PSBs that did not have bound viruses do not form aggregates and remain in solution during magnetic separation. Thus the ECL-intensity obtained from the PSB < ZIKV > MB aggregate is proportional to the concentration of virus in a sample. Figure 4A shows that the ECL intensity of the purified MB-ZIKV-PSB aggregates increase with the amount of virus in the samples. Notably, the ECL response obtained from sample containing 1 PFU of ZIKV is distinguishable from the background (i.e., no virus in the system), indicating that the present detection platform has a detection limit of as low as 1 PFU, which is comparable to RT-PCR (see Fig. 3D). If the concentration of virus in a sample is low, the amount of PSB-virus-MB species formed should be also low. In this case, most of the ECL-signal remains in free (virus-unbound) PSBs that are left in solution during magnetic separation. To confirm these results, we measured the ECL intensity of virus-free PSBs, which we expected to be complementary with the ECL signals obtained from PSB-virus-MB aggregates. Indeed, the ECL signals from the virus-unbound PSBs displayed an inversed relationship with the original amount of ZIKV added to samples (Fig. 4B). This was expected, because each sample used the same amount of PSBs; the more PSB < ZIKV > MB aggregates are formed, the less PSBs remain in the solution. These results also show that there is no loss of ECL-intensity during the assay, confirming that the ECL-intensity of PSB-virus-MB is proportional to the amount of complex formed, hence the amount of virus present in the samples. To analyze further the relationship between ECL-intensity and the amount of virus in the samples, we calculated area under the ECL-curves and plotted the average values against the amount of virus added to the samples. The results show a linear relationship between the virus amount and the ECL-intensity of PSB < ZIKV > MB aggregates as well as free PSBs in the supernatant (Fig. 4C). These results suggest that the ECL-intensity proportionally reflects the amount of viral particles present in samples in a sensitive manner.

To determine whether anti-ZIKV-PSB specifically binds to ZIKV, we tested unrelated (chikungunya virus (CHIKV)) or related (West Nile virus (WNV) and dengue (DENV, serotype 1–4)) mosquito-transmitted viruses with our ECL-based immunoassay. As shown in Fig. 4D, the ECL-intensity of samples containing 10^3^ PFU of CHIKV, WNV, or DENV has a substantially lower ECL signal as compared to ZIKV. The inset of Fig. 4D illustrates the normalized ECL intensity of six viruses compared to ZIKV, and suggests that the detection of ZIKV by an anti-ZIKV-PSB based immunoassay has an acceptable selectivity of ~90%. In addition, we conjugated ECL label loaded PSBs and MBs with a mAb (4G2) that binds to the conserved fusion loop epitope of the E protein of most flaviviruses^46^. These 4G2 conjugated PSB and MB can detect ZIKV (Fig. S1A) and DENV4 (Fig. S1B), suggesting that ECL-based assay can be extended for detection of other viruses. The stability of the ECL-based immunoassay based on PSB < ZIKV > MB system is actually determined by the stability of the RUB/BPO ECL system, because trace amounts of PSB and MB debris as well as bio-substances present in MeCN solution do not interfere the ECL generation. Stable and reproducible ECL signals were observed from the same test solution over different potential cycles and on different testing days.

### ECL-based immunoassay can detect ZIKV in human biological fluids

**Figure 5:**
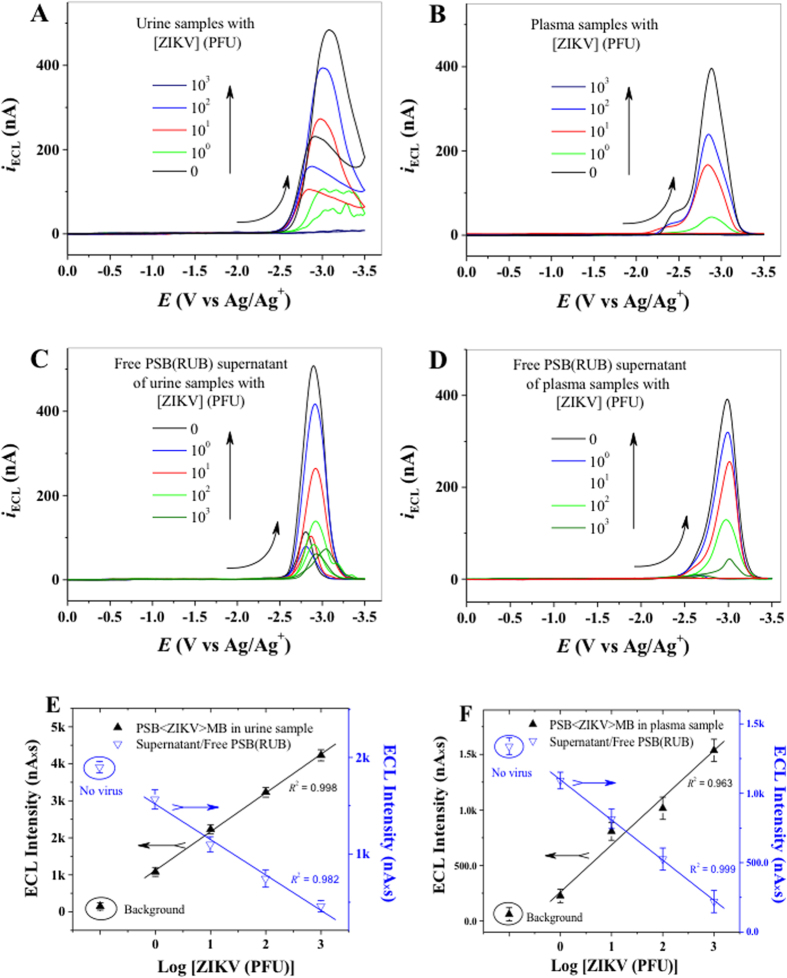
ECL-based immunoassay detects ZIKV in human biofluids. Samples containing different amounts of ZIKV (0–10^3^ PFU) were prepared in human urine or plasma specimens collected from healthy human volunteers. ECL-response from PSB < ZIKV > MB aggregates obtained from (**A**) plasma, and (**B**) urine samples containing different PFUs of ZIKV. ECL-response of virus-free PSB(RUB) that remained in solution after magnetic separation from (**C**) urine and (**D**) plasma samples. A calibration curve of mean ECL intensity of magnetically separated PSB < ZIKV > MB aggregates (upward line, black) and free PSB(RUB)(downward line, blue) that remained in solution after magnetic separation were generated from (**E**) urine and (**F**) plasma samples. No virus control was used to determine background signal. All the experiments were performed in duplicates and repeated at least one time.

ZIKV is present in human blood, urine and other body fluids^16^,^17^,^47^. To test whether our ECL-based immunoassay can detect ZIKV in human body fluids, we collected plasma and urine specimens from healthy human volunteers and spiked in different amount of ZIKV. In these samples, we performed ECL-based immunoassay and measured the ECL-intensity of PSB < ZIKV > MB aggregates. Consistent with the results obtained in PBS, our ECL-based assay detected as few as 1 PFU of ZIKV in 100 µ l of human urine (Fig. 5A) and plasma (Fig. 5B). These results were complemented by measuring the ECL intensity of virus-free PSBs that remain in solution after magnetic separations (Fig. 5C,D), which show a progressive decrease of ECL-intensity with increasing amount of ZIKV present in samples. The area under the ECL curves were plotted against amount of PFUs added to the samples (Fig. 5E,F) and produced a linear curve, suggesting that the ECL-intensity of the PSB-virus-MB aggregates formed is proportional to the amount of ZIKV in the sample. These results establish that the ECL-based immunoassay can detect ZIKV in clinical samples in an ultrasensitive and specific manner.

## Discussion

Mosquito transmitted viruses, such as DENV, Japanese encephalitis virus, and WNV are worldwide public health challenges^48^. Recent emergence of CHIKV^49^ and ZIKV^50^,^51^ has prompted a global public health emergency. Besides mosquito transmission, these viruses also can be transmitted through other routes, such as blood transfusion, organ transplantation, and *in utero*^52^,^53^. For instance, ZIKV virions or RNA can be detected in semen, urine, and saliva, and is categorized as a high-priority infectious agent with the risk of sexual-transmission^19^,^20^,^21^. Importantly, there is no approved vaccine or specific therapy against the most of these mosquito-transmitted viruses. Early and accurate diagnosis of viral infection can reduce the risk of severe consequences, such as DENV hemorrhagic fever, shock syndrome and ZIKV-related developmental defects. Most of the currently available diagnostic methods for viral infection are based on detection of viral antigens, antiviral antibodies, or viral nucleic acid. Although the nucleic acid-based methods are sensitive, they require expensive reagents and instruments. For immunoassays, the performance is often poor due to cross-reactivity with other flaviviruses (e.g. Dengue) and difficulty in clinical correlation. Direct detection of viruses is challenged by requirement of facilities, such as cell culture or electron microscopy, which are not available in most diagnostic laboratories. Thus, a rapid, simple-to-use, sensitive, and cost-effective assay for virus detection in clinical specimen is highly desirable.

An ECL-based detection assay may be ideal for virus detection as it offers several advantages including ultrasensitivity, reproducibility, cost-effectiveness, and rapidness over conventional immunoassays and PCR-based assay. For instances, compared with fluorescence-based methods, ECL assay does not need a light source, which effectively frees the ECL from scattered-light and luminescent impurity interferences. Moreover, the specificity of the ECL reaction associated with an ECL label and a coreactant decreases problems with side reactions (e.g., self-quenching). This is supported by many recent studies^54^,^55^,^56^ in which different immunoassays based on enzyme-linked immunosorbent assay (ELISA), chemiluminescence (CL), ECL, and fluorescence were compared. ECL-based immunoassays had excellent reproducibility and sensitivity over these other assays. We previously demonstrated the feasibility of ECL-based immunoassay for detection of human C-reactive protein (CRP)^30^. Here, we extended this ECL-based immunoassay platform and developed a highly sensitive method for detection of ZIKV. We demonstrate that the PSB conjugated to antibody against viral envelope protein can capture and detect ZIKV in human biological fluids. This assay also could be used to detect ZIKV in mosquito samples in field settings. Moreover, as ZIKV has been suggested to be transmitted by blood transfusion, the ECL-based assay could be developed for blood screening of ZIKV. By utilizing various ECL-emitters (such as RUB, Ru(bpy)_3_^2+^, and DPA) that have easily distinguishable excitation and emission properties^57^, ECL-based immunoassays have multiplexing capacity for simultaneous detection of several types of viruses in a single sample.

In summary, we developed an ultrasensitive, simple-to-use ECL-based immunoassay for specific detection of ZIKV in human body fluids. This proof-of-concept study can be extended to the detection of other viruses or pathogens.

## Experimental Procedures

### Ethics statement and biosafety

All experiments were performed in accordance with guidelines reviewed and approved by the University of Southern Mississippi (USM) Institutional Review Board. Written informed consent was obtained from all human volunteers. All the experiments involving live viruses were performed by the certified personnel in the biosafety level 3 (BSL3) laboratories following the standard biosafety protocols approved by USM Institutional Biosafety Committee.

### Chemicals and reagents

Monoclonal antibody (mAb) against ZIKV (ZV2, IgG2c) specifically recognizes the E protein on ZIKV and was generated after immunization of mice with infectious ZIKV^58^. Mouse mAb specific to flaviviruses (4G2) was produced and purified by culturing hybridoma (HB-112, ATCC) as previously described^59^. Carboxylate polystyrene beads (referred as PSB, 10 μm diameter, 2.6% (w/w) aqueous suspension with approximately 6.5 × 10^4^ beads μl^−1^) and streptavidin-coated super-paramagnetic polystyrene beads (referred as MB, 1.0 μm diameter, 10 mg m l^−1^ aqueous suspension with approximately 9.5 × 10^6^ beads μl^−1^) were purchased from PolySciences Inc. and Dynal Biotech Inc., respectively. Other reagents were purchased from either Sigma or other commercial vendors.

### Preparation and titration of virus stocks

A recent strain of ZIKV (PRVABC59) was obtained from B. Johnson (CDC Arbovirus Branch, Fort Collins CO). CHIKV (LR-OPY1-2006 strain) was provided by R. Tesh (University of Texas Medical Branch). WNV isolate (CT2741) and the four serotypes of dengue viruses (DENV1, DENV2, DENV3, and DENV4) were provided by J. Anderson (Connecticut Agricultural Experiment Station). WNV, ZIKV, and CHIKV were propagated in African green monkey kidney cells (Vero cells, ATCC CCL-81) and DENV (DENV1, DENV2, DENV3 and DENV4) were propagated in C6/36 *Aedes albopictus* cells. All virus stocks were titered in Vero cells (ATCC CCL-81) by plaque assay as previously described^60^.

### ECL label loading and avidin conjugation of PSBs

Loading of ECL-labels to PSBs was performed as previously described^29^,^44^ with some modifications. Briefly, carboxylated PSBs were loaded with RUB by immersing the beads into a saturated solution of RUB (∼0.7 mM) in 5% benzene–95% MeOH (v/v) for 2 h, followed by a series of centrifugation, washing, and drying. Prepared PSBs were designated as PSB(RUB). For avidin conjugation, the PSB(RUB) beads were reacted with a freshly prepared avidin solution (25 μM), activated in a 1-methylimidazole-HCl buffer (0.10 M, pH 7.2) containing 0.10 M EDAC and 0.10 M NHS for 1 h, followed by centrifugation, washing and re-suspension in PBS buffer (pH 7.2). The final solution containing approximately 6.5 × 10^4^ μl^−1^ of avidin conjugated PSB(RUB) beads was stored at 4 °C until use.

### Attachment of biotinylated antibodies to the surface of ECL-label loaded PSBs and MBs

Biotinylation of antibody was performed as described^30^. Biotinylated antibody was immobilized on the surface of ECL-label loaded PSBs by mixing 400 μ l of avidin-coated beads with 1.5 m l of biotinylated antibody (0.05 mg/ml) and rotating the mixture in a Dynal sample mixer at 20 rpm for 1 h. Free antibodies then were removed from the newly formed mAb-PSB(RUB) conjugates by washing four times with PBS and conjugates were re-suspended in 400 μL of PBS (pH 7.2). Likewise, freshly washed MBs corresponding to 100 μl of the original MBs suspension was immersed in 1.5 ml of 0.05 mg/ml of biotinylated antibody, and the mixture was then rotated with a Dynal sample mixer at 20 rpm for 1 h. Newly formed mAb-MB conjugates were separated from the solution mixture with a magnet (Dynal MPC-S), followed by washing and re-suspension in 100 μl of PBS buffer (pH 7.2). The resulting mAb-MB and mAb-PSB(RUB) conjugates were stored at 4 °C until use.

### Sample preparation and ECL-detection of viruses

Viruses were diluted in PBS containing 2% BSA or biological fluids, such as human plasma and urine that were collected form healthy volunteers. Inclusion of 2% BSA minimized the non-specific binding of virus to the surface of beads. Diluted virus solutions (100 μl) containing different PFU of viruses (10^4^ to 10^0^) were mixed with 40 μL of mAb-PSB(RUB) conjugated with virus-specific mAbs and allowed to form conjugates for 45 min at room temperature with gentle shaking in a Dynal mixer at 20 rpm. After washing one time with PBS, 10 μl of mAb-MB solution (i.e., MB/PSB ≈ 36) was added and samples were incubated for 45 min at room temperature with gentle shaking in a Dynal sample mixer at 20 rpm to produce the sandwich-type PSB(RUB)- < Virus > MB aggregates (designated as PSB < ZIKV > MB). The PSB < ZIKV > MB aggregates were fixed in 4% paraformaldehyde (to inactivate virus) and resuspended in 500 μL of PBS, and placed in a Dynal magnetic separator for 5 min. With the tube on the Dynal magnet, the solution was carefully aspirated to remove the unbound fraction. The tube was removed from the magnet and aggregates were resuspended in 500 μl of PBS and again placed in a magnetic separator. This process of magnetic separation and washing was repeated for three times to minimize the possible adsorption of free (virus unbound) PSB(RUB) beads on the wall of the tube, which could produce significant non-specific background signal during ECL measurement.

### ECL and electrochemical measurements

To measure ECL-intensity, aggregates were dissolved in a 2.0 mL solution of 10 mM BPO-0.10 M TBAP in MeCN for optimized ECL measurements (see results for details). A three-electrode system with a 2.0-mm diameter Pt disk as the working electrode, a Pt wire as the counter electrode, and an Ag/Ag^+^(10 mM AgNO_3_ and 0.10 M TBAP in MeCN) as the reference electrode were used. All three electrodes were incorporated in a 5 ml disposable glass vial cell containing assay samples. Before each measurement, the Pt electrode was polished with a 0.3 μm alumina slurry, and all electrodes were cleaned carefully by washing with excess water followed by rinsing with MeCN. The ECL intensities along with the cyclic voltammetry signals (CV) were recorded simultaneously in a home-built ECL instrument as described previously^39^. This instrument consists of a 660A electrochemical workstation (CH Instruments, Austin, TX, USA) combined with a photomultiplier tube (PMT, Hamamatsu R928, Japan) installed under the electrochemical cell. A voltage of −700 V was supplied to the PMT with a high voltage power supply (Model 472A Brandenburg PMT power supply, England). The light signal (as photocurrent) was detected with a high sensitive Keithley 6514 electrometer (Keithley, Cleveland, OH, USA) and converted to voltage (in ±2 V) that was collected to the electrochemical workstation computer. Unlike the commercially available BioVeris ECL system, where flow-cell based ECL detection is used; our ECL instrument uses conventional electrochemical cells. For cathodic ECL measurements, the testing solutions were pre-degassed with ultra-high purity of nitrogen (Airgas South, Hattiesburg, MS) for 7–10 min. All measurements were conducted at a temperature of 23 ± 2 °C.

### Microscopy

ECL label loaded PSBs and PSB-virus-MB aggregates were imaged in a Zeiss LSM510 META confocal microscope (Carl Zeiss, NY).

### Real time-quantitative PCR (RT-qPCR)

ZIKV particles attached on the surface of beads (i.e., PSB < ZIKV > MB) were quantified by RT-qPCR, as described previously^60^. Briefly, PSB < ZIKV > MB complexes prepared with different concentrations of ZIKV were treated with TRI-reagent (Molecular Research Center) to lyse viral particles, and viral RNA was isolated after adding 1 μg of tRNA (ThermoFisher Scientific) as carrier. The complementary DNA (cDNA) was synthesized using the iSCRIPT cDNA synthesis kit (Bio-Rad) and RT-qPCR assays were performed in a CFX96 Real-Time system (Bio-Rad) using iTaq universal probe supermix (Bio-Rad). Primers (Forward, GGAGTCAGCAATAGGGACTTTG; Reverse, CGGTTTGTCCTGTGCCATTA) and probe (FAM/AGGTGGGAC/ZEN/TTGGGTTGATGTTGT/3IABkFQ) specific to the Envelope gene of ZIKV were designed and synthesized by Integrated DNA Technologies.

### Western Blotting

Antibody conjugation to beads was analyzed by immunoblotting as previously described^60^. Briefly, the beads were treated with Laemmli sample buffer (Bio-Rad) and proteins were separated by 10% SDS-polyacrylamide gel electrophoresis, followed by transfer to a nitrocellulose membrane (Bio-Rad). Heavy chain and light chain of antibody were detected by probing the membrane with horseradish peroxidase conjugated goat anti-mouse IgG antibody (Jackson Immunoresearch). Images were acquired in a ChemiDoc MP system (Bio-Rad) by using SuperSignal West Pico Chemiluminiscence Substrate (Thermo Scientific).

## Additional Information

**How to cite this article**: Acharya, D. *et al.* An ultrasensitive electrogenerated chemiluminescence-based immunoassay for specific detection of Zika virus. *Sci. Rep.* **6**, 32227; doi: 10.1038/srep32227 (2016).

## Supplementary Material

Supplementary Information
