# Supplementary material for: An ultrasensitive electrogenerated chemiluminescence-based immunoassay for specific detection of Zika virus
Source: Sci Rep. 2016 Aug 24;6:32227. doi: 10.1038/srep32227 (PMC4995374; doi:10.1038/srep32227)
Supplement: Supplementary Information [file srep32227-s1.pdf]

Supplementary Information

An ultrasensitive electrogenerated chemiluminescence-based immunoassay for specific detection of Zika virus

Dhiraj Acharya[1§], Pradip Bastola[2§], Linda Le[1], Amber M. Paul[1], Estefania Fernandez[3], Michael S. Diamond[3], Wujian Miao[2*] and Fengwei Bai[1*]

Fig. S1

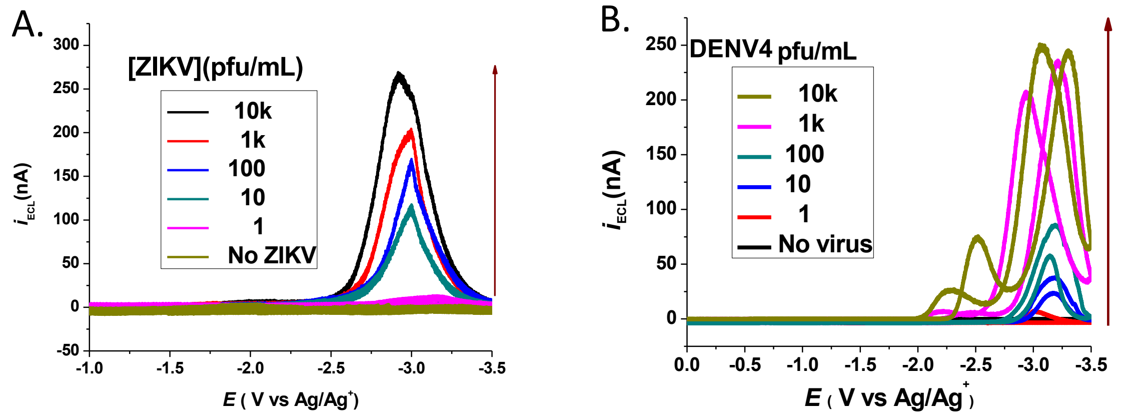

Supplemental figure legend:

Figure S1. ECL-based method has wide applications in detection of viruses. ECL label loaded PSBs and MBs were conjugated with a flavivirus cross-reactive mAb (4G2) that detects most flaviviruses. Samples containing different amounts (0-10^4 PFU) of ZIKV or DENV in PBS containing 2% BSA were allowed to form PSB<ZIKV>MB aggregates by reacting with anti-4G2-PSB(RUB) and anti-4G2-MB. ECL-response from PSB-ZIKA-MB (**A**) and PSB-DENV4-MB (**B**) aggregates obtained from samples containing different concentrations of respective viruses. All the experiments were performed in duplicates and repeated at least one time.
